# A Plea for a More Amicable Relationship and Greater Sociability between Physicians

**Published:** 1888-11

**Authors:** T. B. Greenley


### A PLEA FOR A MORE AMICABLE RELATIONSHIP AND GREATER SOCIABILITY BETWEEN PHYSICIANS.

By T. B. GREENLEY, M. D.

The question is frequently asked by the laity, Why is it that doctors get along so badly with each other?

This question grows out of the fact that physicians, in little towns more particularly, are often at loggerheads, ignoring all social intercourse, and occasionally become belligerant. Such a state of affairs between physicians in a neighborhood is to be greatly lamented, and proves to be a great disadvantage, not only to themselves but to the people, as it is not uncommon for the latter to take sides with their favorite physician even to sharing with him his acrimonious feelings. Owing to such an unpleasant feud, when sickness occurs and consultation is wanted, the partisans of one doctor will not have the other even if he would agree to consult, which he generally refuses to do; hence, a consulting physician must be had from a distance, both to the cost of the sick man as well as the discredit of the local physicians.

This kind of trouble between physicians hardly ever results from want of professional knowledge on either side, but from other causes, and is usually of a trivial character. Sometimes it may have its origin in what some tattler or gossip lover may have heard as coming from one physicians speaking slightly of another, and perhaps without any foundation, or, if any, greatly magnified. The writer has had experience in this particular on more than one occasion. Quite recently he was called to see a lady in the process of abortion, who had been under the care of a physician at some distance. On her return home it got to the ears of the doctor that I should have said he had been guilty of malpractice in her case, upon which he wrote me a very insulting note. Of course nothing had been said by me derogatory to his medical standing. Now as this man had known me from his student days, and knew that I had always treated him courteously, and, I might say, assisted him in word and deed, it would have been a small matter for him to have written me and inquired in a friendly manner whether I had said so and so respecting him. In another instance, also of recent occurence, I was called to see a child in the absence of the attending physician. I prescribed and left a note for the doctor, as I always do in such cases. Not long after I heard that the mother of the child had told the neighbors that I had said the child had not been treated correctly, and that young doctors knew nothing about treating children.

The next time I met the young doctor, instead of a quarrel or fight, we had a big laugh over the matter. He had also known me from boyhood, and had more sense than to believe I had made any such remark.

Physicians are, perhaps, the most sensitive class of men in the world, and brook slighting remarks with little patience; this more particularly in regard to their professional acquirements, as it is natural for every doctor to think he is well-qualified. More especially is this so with the young doctor just graduated. It should be a rule with every physician to speak well of his brother doctor, if he speaks of him at all; and if he cannot conscientiously speak in his favor, he should not speak against him, for the least insinuation to his detriment is apt to reach him greatly magnified. Professional jealously in many instances, serves as a ground-work for unpleasant feelings between medical men. Should a neighbor doctor get more calls than we do, it is no good reason for unkind feelings on our part, especially if he does not obtain his practice by trickery or detraction of us. If he observes the ethical code and is a gentleman, we are bound, as honorable members of the profession, to treat him with gentlemanly courtesy.

We must recollect that it is not possible that all members ot the profession should succeed at once in obtaining a lucrative practice; and if it should fall to our lot to fail in this particular, we should not cast any blame on any one else, but charge it to fate and wait with patience for our time to come. But in the meantime we should not expect it to come ro us unless we are prepared for it, and I can assure the young members that they will not be prepared for it if they spend their time idly at the street corners, post-office, or depot, gossiping with loungers. The proper place for a physician when not engaged in professional duties, is in his office among his books and journals It is here he can prepare himself to meet the calls of the sick and to acquit himself with credit as a physician.

There are very few men in the profession who will not finally succeed in their calling if they pursue the proper course in every particular. Of course, it is understood that when speaking of physicians we mean regular members of the fraternity, and we also mean *gentlemen*, for I think, above all professions, divinity not excepted, physicians should be gentlemen. As before remarked, all should not expect to succeed at once, but those who do not must bide their time with patience.

We should remember the remark of Daniel Webster, when a young man asked his advice relative to studying law. He said, "There is plenty of room at the top, but the bottom is crowded." This properly illustrates the condition of our profession—there is plenty room in the upper stories, but the bottom is crowded. But, while many of us are at the bottom and endeavoring to ascend, we should not envy those above us if they have arrived at their eminence by true merit and not by intrigue; but, on the contrary, we should award them due credit for their attainments, and strive to emulate their virtues and industry. And those who have been so fortunate as to have mounted high up the ladder should look down kindly on those at the foot, who, with meritorious endeavors, are trying to ascend, and by encouraging words assist them up. I think every deserving young physician should be kindly taken by the hand and encouraged by the older members of the profession. But, unfortunately, the contrary is too often practiced. The young man is frequently not only ignored, but regarded by the old doctor and spoken of as a quack or ignoramus, when at the same time, perhaps, he may be far superior in scientific attainments to his elder *confrere.*

When we regard the matter of kindness that should be extended to the young doctors by the elder members of the profession in its proper light, humanity and patriotism both compel us to the performance of that duty. If we reflect for a moment we are impressed with the fact that those who are at the bottom, and earnestly and honorably striving to ascend, must sooner or later take our places. Therefore it becomes us to do all in our power to induce them to qualify themselves in the best manner possible for the successful management of disease, while it is patriotic to wish our successors ability to maintain the dignity, honor and renown which the Commonwealth already enjoys in medical science.

The profession of medicine, when acquired by honorable men, should be a tie to bind them together in a friendly, social manner, as the sign that cemented the friendship of Jonathan and David. Our profession, in a word, should make brothers of us, no difference whether we stand high up on the ladder or at the bottom; whether we are old in the profession or newly-fledged graduates. It is gratifying to look back a quarter a century and comtemplate the position occupied by the medical profession during the unhappy civil war. But very few on either side became active partisans, and friendly intercourse, as a rule, was maintained between the members of the profession of the two sections. I have thought that to some extent peace was effected a little sooner by the friendly relation maintained by the members of the medical profession and those of the Masonic fraternity of the North and South.

In conclusion, I would like to say a word respecting a few of the great men of the profession of our State—those who by force of talents and merit ascend the ladder of fame and arrive at the topmost round. It is quite common to eulogize the great dead, and even to erect monuments to their memory, which is a very laudable practice, and one it is to be hoped will be continued for all time. It cannot be denied that the fame acquired by the members of the profession of Kentucky is equal to, if not greater, than that of any other State. In order to illustrate this fact we have only to refer to our McDowell, our Brashear, and our Dudley—the first to originate an operation that has given thousands of years of life to suffering women; the second also the originator of a capital operation, requiring great genius and dexterity, and which is now practiced the world over for the benefit of the afflicted; and the third, the most expert and successful lithotomist of his time. Were we not to hold the memory of these men with pride, gratitude, and respect, we should be recreant to the proper instincts of our profession, as well as destitute of that patriotism which every Kentuckian should possess.

But we can call to mind other great men of our State who hold in no small measure our veneration and respect. Who among us can ever have blotted from his memory the names of Gross, of Yandell, of Drake, of Caldwell, and many other great lights of medicine who wrote their name high on the scroll of fame, doing credit to themselves and honor to their State. It causes the professional heart to thrill with patriotic pride when the names of our great men are called to mind, and we are always ready to award the due meed of praise to their memories.

I have often thought that it might be well to change, to some extent, our mode awarding praise to the great men of our profession. Let us not confine ourselves to bestowing posthumous praises and honors, but award them, in part at least, *ante mortem.* To some extent this was done in the case of our revered and illustrious Gross. He was termed the Nestor of Surgery in America, besides having other honors conferred on him both in this country and Europe. And no man ever wore his honors with more dignity and circumspection.

Nothing could be more gratifying and pleasing to one's senses than to have his merits recognized and appreciated by his fellow-workers in medicine, and I think it would be a happy thing to manifest some of our respect and appreciation for the great men of our profession while living, and not wait to commemorate their virtues after their work is ended.—*Amer. Prac. and News.*

